# Pro-inflammatory effects of crystalline- and nano-sized non-crystalline silica particles in a 3D alveolar model

**DOI:** 10.1186/s12989-020-00345-3

**Published:** 2020-04-21

**Authors:** Tonje Skuland, Marit Låg, Arno C. Gutleb, Bendik C. Brinchmann, Tommaso Serchi, Johan Øvrevik, Jørn A. Holme, Magne Refsnes

**Affiliations:** 1 Section of Air Pollution and Noise, Department of Environment and Health, Norwegian Institute of Public Health, PO Box 4404 Nydalen, N-0403 Oslo, Norway; 2 Environmental Research and Innovation (ERIN), Luxembourg Institute of Science and Technology (LIST), Belvaux, Grand Duchy of Luxembourg, Luxembourg; 3 Department of Occupational Medicine and Epidemiology, National Institute of Occupational Health, Oslo, Norway; 4 Department of Biosciences, Faculty of Mathematics and Natural Sciences, University of Oslo, Oslo, Norway

**Keywords:** Silica nanoparticles, Crystalline silica, Inflammation, 3D tri-cultures, Lung epithelial cells, THP-1 macrophages, Endothelial cells

## Abstract

**Background:**

Silica nanoparticles (SiNPs) are among the most widely manufactured and used nanoparticles. Concerns about potential health effects of SiNPs have therefore risen. Using a 3D tri-culture model of the alveolar lung barrier we examined effects of exposure to SiNPs (Si10) and crystalline silica (quartz; Min-U-Sil) in the apical compartment consisting of human alveolar epithelial A549 cells and THP-1-derived macrophages, as well as in the basolateral compartment with Ea.hy926 endothelial cells. Inflammation-related responses were measured by ELISA and gene expression.

**Results:**

Exposure to both Si10 and Min-U-Sil induced gene expression and release of CXCL8, interleukin-6 (IL-6), tumor necrosis factor-α (TNF-α), interleukin-1α (IL-1α) and interleukin-1β (IL-1β) in a concentration-dependent manner. Cytokine/chemokine expression and protein levels were highest in the apical compartment. Si10 and Min-U-Sil also induced expression of adhesion molecules ICAM-1 and E-selectin in the apical compartment. In the basolateral endothelial compartment we observed marked, but postponed effects on expression of all these genes, but only at the highest particle concentrations. Geneexpressions of heme oxygenase-1 (HO-1) and the metalloproteases (MMP-1 and MMP-9) were less affected. The IL-1 receptor antagonist (IL-1RA), markedly reduced effects of Si10 and Min-U-Sil exposures on gene expression of cytokines and adhesion molecules, as well as cytokine-release in both compartments.

**Conclusions:**

Si10 and Min-U-Sil induced gene expression and release of pro-inflammatory cytokines/adhesion molecules at both the epithelial/macrophage and endothelial side of a 3D tri-culture. Responses in the basolateral endothelial cells were only induced at high concentrations, and seemed to be mediated by IL-1α/β released from the apical epithelial cells and macrophages.

## Background

Silica dioxide is a non-metallic oxide which exists in nature both in crystalline and non-crystalline (amorphous) forms, and both forms have a wide range of technical applications. Amorphous silica particles, including silica nanoparticles (SiNPs), have unique properties making them among the three most produced nanoparticles (NPs) world-wide. They are extensively used in agriculture, food, and various consumer products [1]. Exposure to crystalline silica (quartz) in the micro-meter size-range, is known to induce airway diseases, including silicosis, chronic obstructive pulmonary disease (COPD), chronic bronchitis and cancer [2, 3], with severity of effects varying dependent on particle source [4]. Non-crystalline silica in the submicro-meter size-range seems to be less toxic since they are not persistent in the airways compared to the crystalline silica particles [5–7], and is presumably not involved in chronic toxicity. However, increasing use of non-crystalline SiNPs may raise concerns that these NPs may elicit more harmful effects than their sub-micrometer counterparts, and in particular for acute toxicity [1, 8]. Crystalline silica particles and SiNPs are known to be taken up by different mechanisms and cleared via different efficiency by macrophages, dependent on particle size and cell type [8–10]. Both types of particles induce cytotoxic, genotoxic and pro-inflammatory acute responses [1, 4, 8]. In previous studies we have investigated SiNP-induced cytotoxicity, cytokine gene expression and release as well as underlying signalling pathways involved, in lung epithelial cells and macrophages in mono-cultures [11–14].

The toxicity of crystalline silica seems to be dependent on silanol groups at the particle surface [15], and is by large considered to arise from activation of inflammatory responses in the pulmonary tissue. Central is the profound cytotoxicity observed in macrophages, where phagocytosed crystalline silica particles cause rupture of phagolysosomes, leakage of lysosomal constituents into the cytoplasm activating the inflammasome (NLP3). This leads to activation of caspase-1, and subsequent cleavage, activation and release of the highly inflammatory cytokine IL-1β along with release of a number of other pro-inflammatory alarmins [16, 17]. However, pulmonary inflammation induced by crystalline silica also involves other key target cells and in particular pulmonary epithelial cells. Also in the epithelial cells, crystalline silica-induced pro-inflammatory responses appear to involve inflammasome activation [18]. These inflammatory responses do not appear to be restricted to crystalline particles. Also different non-crystalline (amorphous) silica particles of nano-, submicro- and micro-sizes may induce release of IL-1β from cells via activation of the inflammasome [12, 19, 20]. In vitro studies show that both larger crystalline particles and SiNPs may induce inflammation-related responses, including release of pro-inflammatory cytokines (IL-6 and CXCL8). Furthermore, SiNPs appear to be far more potent than submicro-sized (0.1–1 μm) amorphous particles, estimated per mass [12, 21–24]. A central question is whether the high surface area to mass ratio of SiNPs compensates for lower surface reactivity, compared to crystalline silica, and thereby is a driver of pulmonary toxicity. During pulmonary inflammation, inflammatory mediators from macrophages and epithelial cells activate endothelial cells, which may amplify the inflammatory signal, releasing further inflammatory mediators into the circulation and increase the expression of adhesion molecules on the endothelial surface, to activate and attract immune cells to the site of inflammation [25]. Thus, responses of the pulmonary endothelial cells are also central in development of lung disease, COPD. Endothelial dysfunction in the lung during COPD shares pathological mechanisms with arterial and pulmonary hypertension, atherosclerosis and systemic inflammation [26, 27]. Importantly, the release of inflammatory mediators into circulation is an integral part of any inflammatory response. However, acute excessive or chronically elevated levels of circulating cytokines due to high-dose or continuous exposures to particulates, may pose a risk of onset of inflammatory reactions in secondary organs and subsequent tissue damage. In particular, pulmonary inflammation may lead to development or exacerbation of cardiovascular disease [26]. In line with this, both occupational exposure and experimental studies in mice suggest that silica-exposure may result in development of cardiovascular outcomes [28–30]. As crystalline silica particles are too large to cross the alveolar barrier and enter circulation to any considerable degree, the cause of these effects are most likely due to release of inflammatory mediators. Crystalline silica may also cause vascular remodelling in the airways, which may contribute to development of pulmonary hypertension, an irreversible condition associated both with, and complicating, silicosis and COPD [31–33]. IL-1α and -β are suggested to be among the key factors involved in development of both pulmonary and cardiovascular effects from silica exposure [30]. IL-1α and IL-1β activate a common IL-1 receptor (IL-1R), and seem via inflammation to be involved in a range of pathological processes [34–36]. In addition, TNF-α appears to be critical in the onset of pro-inflammatory signalling in silica-induced pathologies [37, 38]. In contrast to larger crystalline silica particles that presumably exert their effects via such pro-inflammatory mediators, small SiNPs may translocate across the airway barrier to the systematic circulation, and potentially exert direct effects in secondary organs [39, 40].

The last years increasing attention has been paid to: a) develop in vitro systems for high throughput screening for studying NPs [41] b) identify adverse outcome pathways (AOP) and their critical molecular initiating events (MIEs) for NPs [42] c) develop relevant 3D co-cultures better mimicking the in vivo situations [43–47]. In several studies THP-1 monocytes or PMA-differentiated THP-1 monocytes (THP-1 macrophages), have been included in the apical or basolateral compartment [43, 48]. The apical compartment in such tri-culture models has been exposed to crystalline silica [49, 50], particulate matter [51, 52], diesel exhaust particles (DEP) [43, 53, 54] as well as NPs, including SiNPs [53] and silver (Ag) NPs and nanowires [48, 55, 56]. The most common biological endpoints include barrier function, cytotoxicity and pro-inflammatory responses in the apical and basolateral compartment, with some studies reporting endothelial responses [49, 53–57], others not [53, 55]. Both submerged and air-liquid interphase (ALI) exposures have been applied [46, 55]. Our previous studies have shown that crystalline silica (Min-U-Sil) and amorphous silica particles from nano- to micrometer sizes are inducing release of IL-1β from macrophages via an inflammasome mechanism [12]. We have also shown that SiNPs may also increase the release of not only IL-1α/β from lung epithelial cells but also CXCL8, IL-6 and TNF-α [58].

In the present study, we have examined the potential of SiNPs (Si10), and micrometer-sized crystalline silica particles (quartz; Min-U-Sil), to induce pro-inflammatory responses in a 3D tri-culture cell model, mimicking the lung-blood barrier, as introduced by Klein and co-workers [43, 44]. The culture is consisting of apical PMA-differentiated THP-1 cells and A549 cells in contact culture, separated from basolateral endothelial cells (Ea.Hy926) by a micro-porous membrane (1 μm pores). The main hypothesis is that both Min-U-Sil and Si10 particles may trigger the release of IL-1α/β in the apical compartment of the co-culture cell model, and that these mediators may cross the filter/cell barrier and thus induce gene expression in the endothelial cells. Finally, we hypothesize that the endothelial gene expression responses in the basolateral compartment are delayed compared to the gene expression responses of the apical macrophages/epithelial cells.

## Results

### Cytotoxic and pro-inflammatory responses to silica particles in 3D co-culture

The apical compartment of a 3D tri-culture cell model was exposed to Si10 (0–48 μg/cm^2^) and Min-U-Sil (0–192 μg/cm^2^) for 2–20 h. Cytotoxicity, gene expression and release of pro-inflammatory cytokines were measured. In addition, gene expression of some cell adhesion molecules, metalloproteases and heme oxygenase (HO-1) were measured.

#### Cytotoxicity

No cytotoxicity was observed even at the highest concentrations tested, neither in the apical nor basolateral compartment as measured by AlamarBlue (Fig. S1 in Appendix).

#### IL-6 and CXCL8 release

**Fig. 1:**
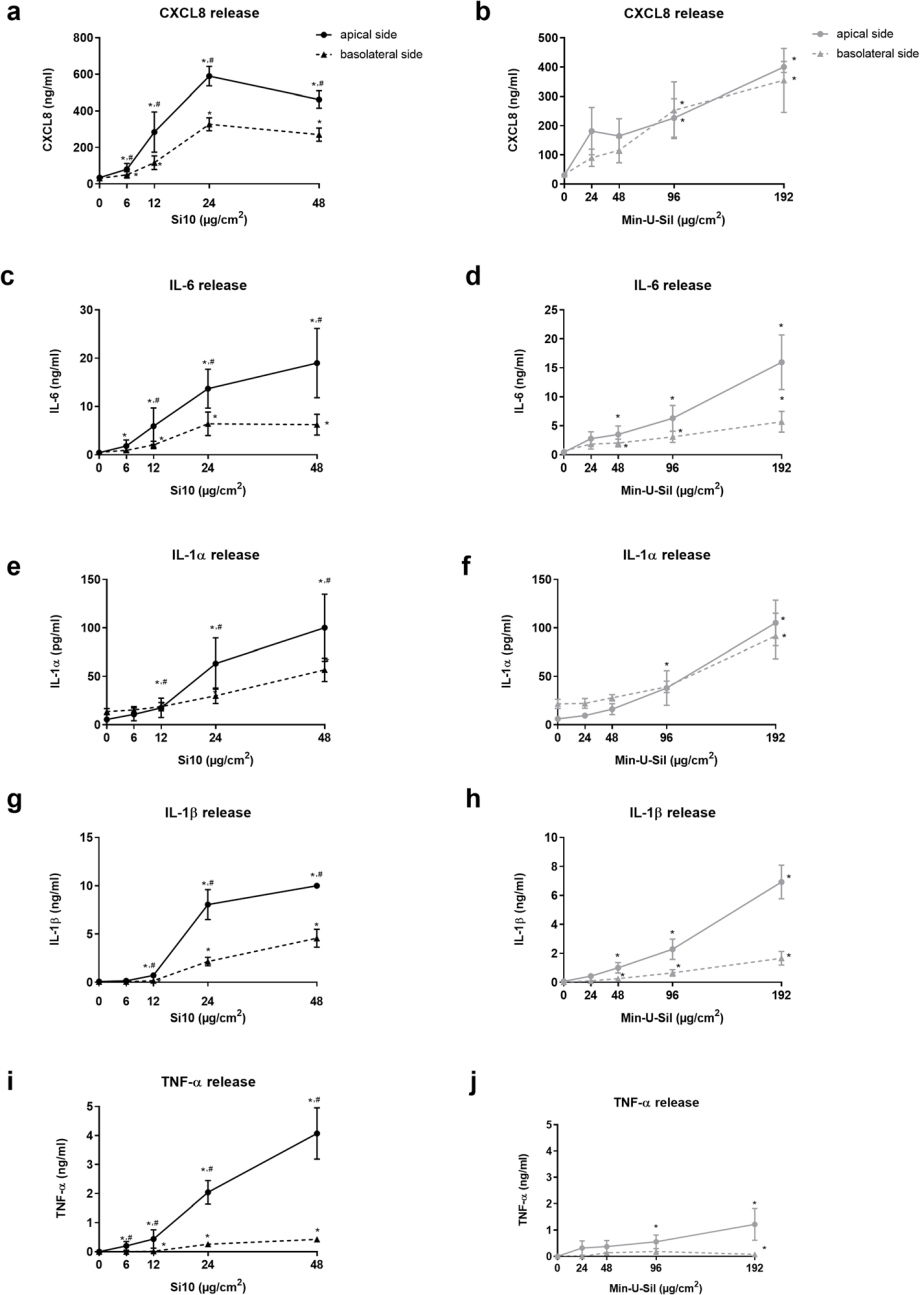
Concentration-dependent release of cytokines after Si10 and Min-U-Sil exposure in 3D tri-culture. The 3D tri-culture consisted of THP-1 macrophages and A549 cells in the apical compartment and Ea.hy 926 endothelial cells in the basolateral compartment as described in Material and Methods. The figure shows cytokine levels in the apical and the basolateral compartment after 20 h exposure to Si10 (0–48 μg/cm^2^) and Min-U-Sil (0–192 μg/cm^2^). Cytokine levels were determined by ELISA, and data represent the mean +/− SEM of 5 independent experiments. *Statistical significant difference from control, #statistical significant difference from basolateral side, *P* < 0.05

Levels of several pro-inflammatory cytokines were analysed in medium from the apical as well as the basolateral compartment, after 20 h exposure to Si10 or Min-U-Sil. Si10 exposure at concentrations from 6 to 48 μg/cm^2^ induced IL-6 and CXCL8 release in both compartments (Fig. 1a, c). Upon exposure to 24 μg/cm^2^ Si10, the amount of IL-6 increased from 0.5 to 13.7 and 6.4 ng/ml in the apical and basolateral compartment, respectively (Fig. 1c). Release of CXCL8 increased from 30 to 600 and 325 ng/ml, respectively (Fig. 1a). Effects of Min-U-Sil were observed at higher concentrations (96 and 192 μg/cm^2^ for CXCL8; 48–192 μg/cm^2^ for IL-6) (Fig. 1b, d). Upon exposure to 192 μg/cm^2^ of Min-U-Sil the releases of IL-6 and CXCL8 were of the same magnitude as for 24 μg/ml of Si10 (Fig. 1a,c).

#### IL-1α and IL-1β release

Si10 and Min-U-Sil increased levels of IL-1α and IL-1β in both compartments of the 3D tri-cultures (Fig. 1e-h). IL-1α levels were quite low compared to IL-1β (approximately 100-fold lower), only 0.060 ng/mL in the apical and 0.030 ng/mL in the basolateral compartment after exposure to 24 μg/cm^2^ of Si10 (Fig. 1e). IL-1β levels were about 8 and 2.1 ng/mL in the apical versus basolateral compartment after exposure to 24 μg/cm^2^ Si10 (Fig. 1g). Exposure to 192 μg/cm^2^ of Min-U-Sil led to IL-1α release of 0.105 ng/mL and 0.090 ng/mL in the apical and basolateral compartment, respectively. The IL-1β release was 7 ng/mL and 2 ng/mL in the apical and basolateral compartment, respectively (Fig. 1f, h). At the lower concentrations of both Si10 (12 μg/cm^2^) and Min-U-Sil (48 μg/cm^2^) the IL-1β levels were significantly increased, but with levels less than 1.0 and 0.2 ng/ml in the apical and basolateral compartment, respectively, showing that the responses are highly concentration-dependent.

#### TNF-α release

Exposure to Si10 and Min-U-Sil also led to an increase in TNF-α levels in both compartments. As observed for the other cytokines, Si10 was found to be more potent than Min-U-Sil (Fig. 1i, j). Upon exposure to 24 μg/cm^2^ Si10, TNF-α release was 2 and 0.26 ng/ml in the apical and basolateral compartment, respectively (Fig. 1i). Upon exposure to 192 μg/cm^2^ of Min-U-Sil, TNF-α release was 1.2 and 0.08 ng/ml in the apical and basolateral compartment, respectively (Fig. 1j).

#### Cytokine responses to Si10 in endothelial cell monocultures

A critical question is whether Si10 may translocate across the cell barrier in the 3D co-cultures and trigger direct pro-inflammatory responses in the endothelial cells. To elucidate this we examined the potential of Si10 to directly induce cytokine release in Ea.hy 926 endothelial cells in mono-cultures. With the assumption that all the Si10 particles could pass the epithelial/ macrophage barrier on the insert in the 3D co-culture, we used an equivalent concentration range as in the co-culture. The cells showed marked concentration-dependent IL-1α, CXCL8 and IL-6 responses upon Si10 exposure, but no detectable IL-1β and TNF-α responses. For low concentrations of Si10, no or only small cytokine responses were observed (Fig. S2 in Appendix).

### Expression of inflammation-related genes

#### Pro-inflammatory cytokines

**Fig. 2:**
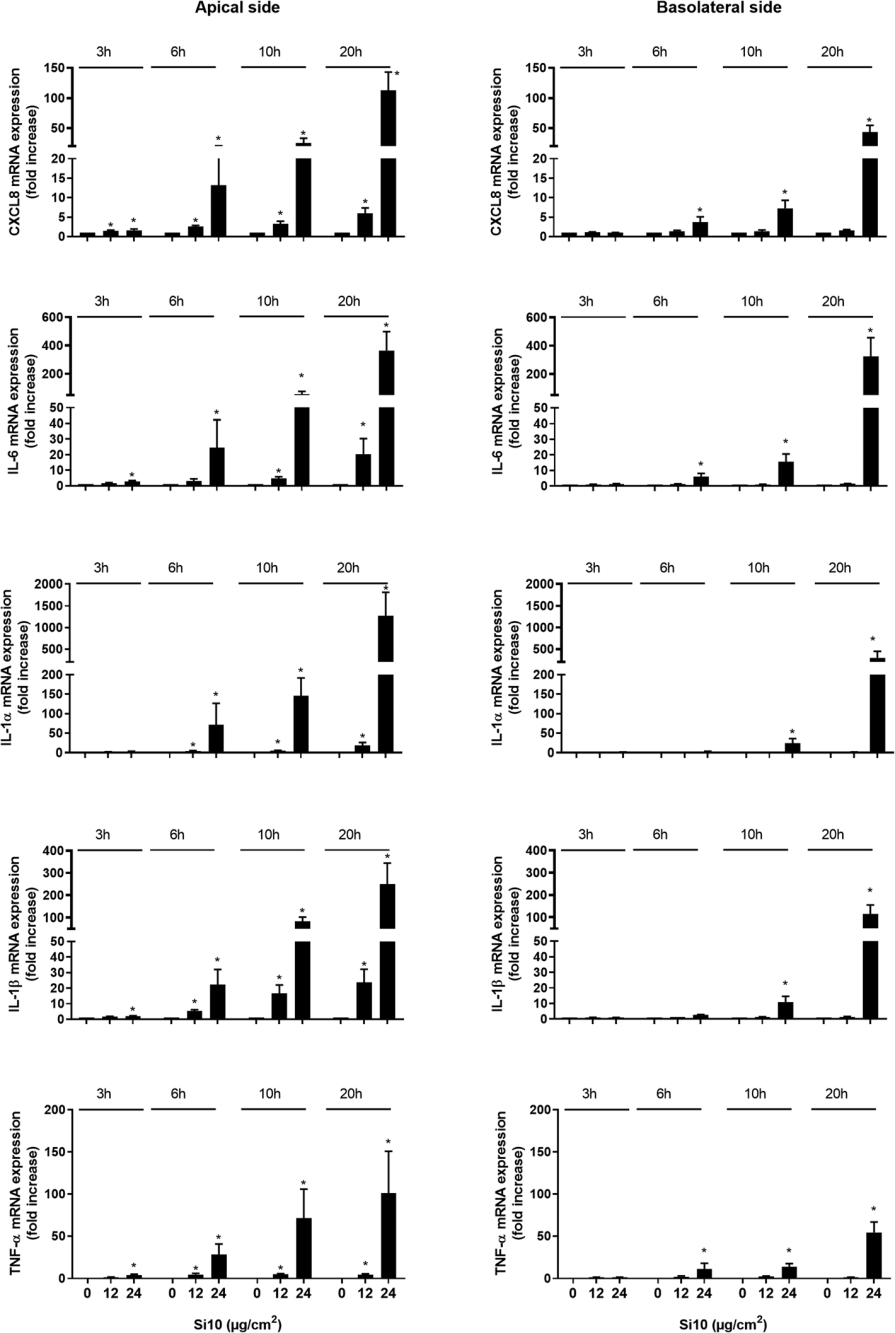
Time-dependent CXCL8, IL-6, IL-1α, IL-1β and TNF-α gene expression on the apical and basolateral side of 3D tri-cultures after Si10 exposure. The figure shows cytokine expression 3, 6, 10 and 20 h after Si10 exposure (12 and 24 μg/cm^2^). Gene expression was determined by qPCR, and data presented as fold increase compared to control. Data represent the mean +/−SEM of 3–5 independent experiments.*Statistical significant difference from control at the same time-point *P* < 0.05

**Fig. 3:**
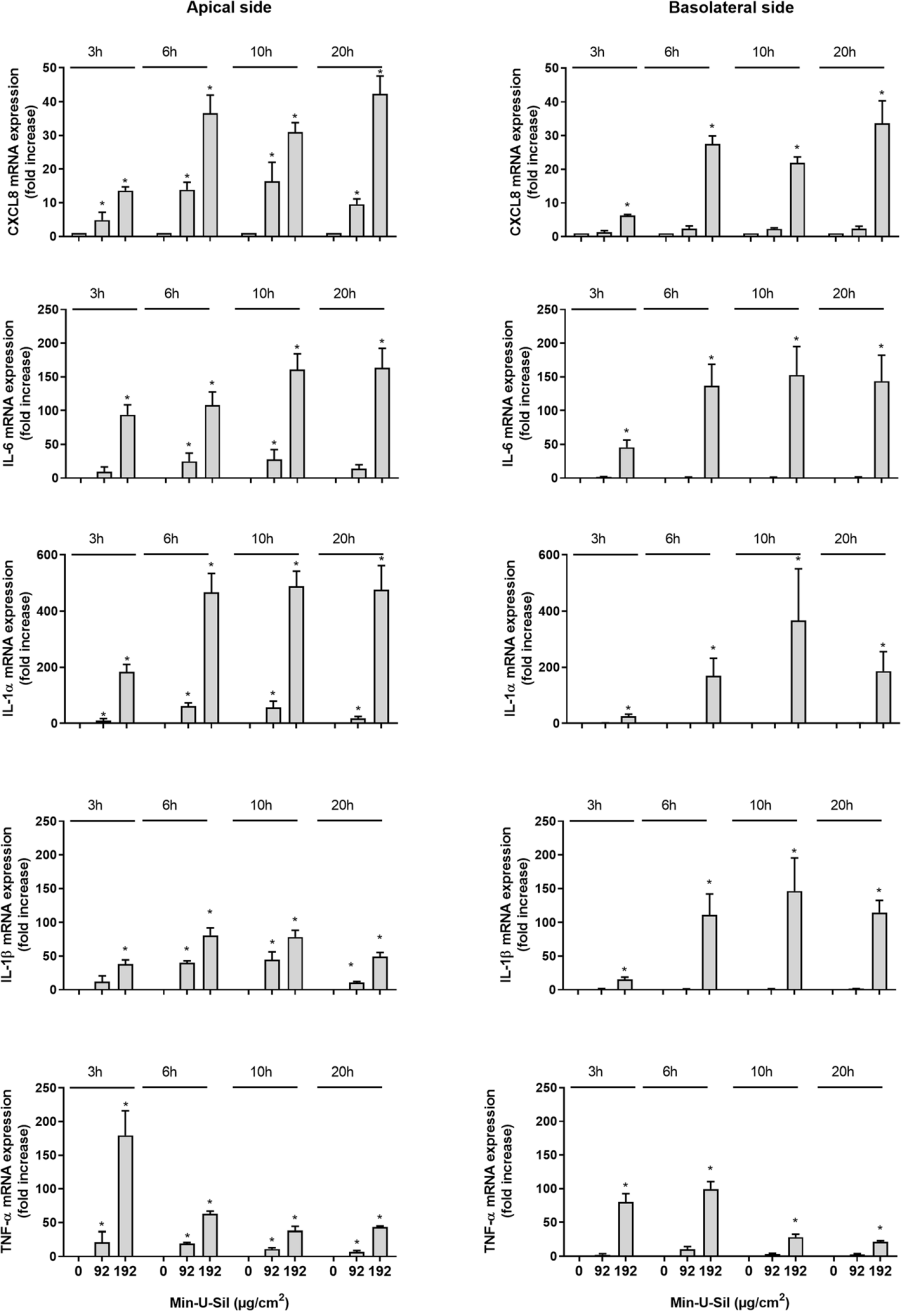
Time-dependent gene expression of CXCL8, IL-6, IL-1α, IL-1β and TNF-α on the apical and basolateral side of 3D tri-cultures after Min-U-Sil exposure. The figure shows gene expression 3, 6, 10 and 20 h after exposure to Min-U-Sil (96 and 192 μg/cm^2^). Gene expression was determined by qPCR, and presented as fold increase compared to control. Data represent the mean +/− SEM of 3–5 independent experiments. *Statistical significant difference from control at the same time-point *P* < 0.05

Gene expressions of CXCL8, IL-6, IL-1α, IL-1β and TNF-α were assessed on the apical and basolateral side of the filter membrane after 3, 6, 10 and 20 h of exposure to 12 and 24 μg/cm^2^ of Si10 (Fig. 2) and 96 and 192 μg/cm^2^ of Min-U-Sil (Fig. 3). The lowest concentrations of Si10 particles (12 μg/cm^2^) increased expression of CXCL8, IL-6, IL-1α, IL-1β and TNF-α in the apical epithelial cells/macrophages from 6 to 20 h, whereas no up-regulation was observed in the basolateral endothelial cells. The highest concentrations of Si10 (24 μg/cm^2^), induced a more marked up-regulation of CXCL8, IL-6, IL-1α, IL-1β and TNF-α expression in the apical compartment from 3 to 6 h, with approximately 110-, 350-, 1200-, 250- and 100-fold increases, respectively at 20 h. Furthermore, at this Si10 concentration, a time-dependent up-regulation was observed from 6 h in the gene expression of the cytokines also in the basolateral endothelial compartment, with approximately 40-, 300-, 300-, 100- and 50-fold increase, respectively, at 20 h (Fig. 2).

For Min-U-Sil a similar pattern was observed as for Si10. The lowest concentration (96 μg/cm^2^) induced a significant up-regulation in the gene expression of CXCL8, IL-1α and TNF-α in apical compartment already 3 h after exposure, and the gene expressions continued to rise in the period from 6 to 10 h (with the exception for TNF-α). At the higher concentration (192 μg/cm^2^) responses were even more pronounced upon exposure in the apical compartment. At 20 h up-regulations of CXCL8, IL-6, IL-1α, IL-1β and TNF-α gene expressions were approximately 40-, 150-, 500-, 50- and 40-fold, respectively. With respect to the basolateral compartment, no significant increases in gene expressions for the different cytokines were observed after exposure to the lowest concentration of Min-U-Sil. However, upon exposure to the highest concentration marked up-regulations were observed on the basolateral side with significant but low responses at 3 h. After 20 h a 30-, 150-, 180-, 115-, 20-fold increases were measured for CXCL8, IL-6, IL-1α, IL-1β and TNF-α, respectively (Fig. 3).

#### Intercellular adhesion molecules

**Fig. 4:**
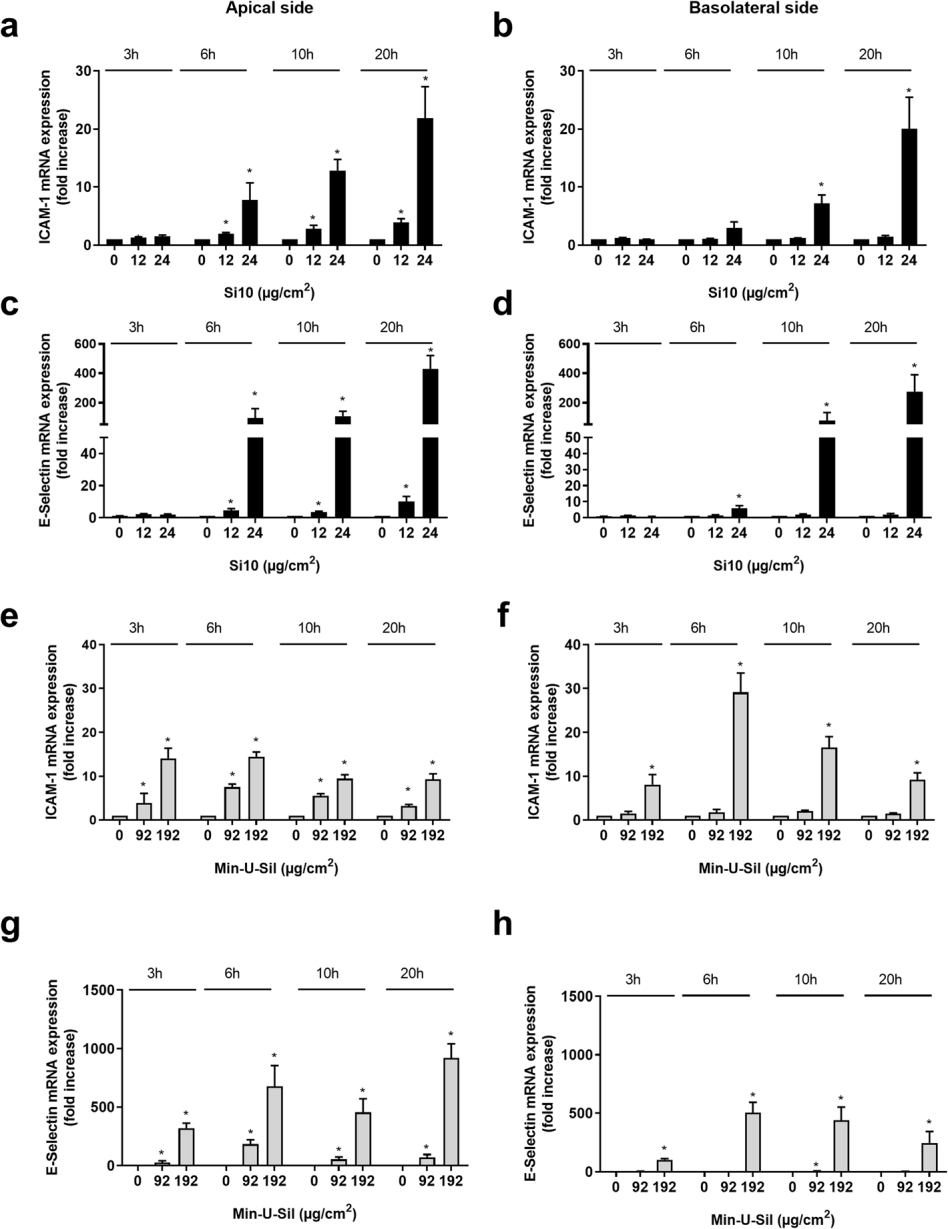
Time-dependent gene expression of ICAM-1 and Selectin E on the apical and basolateral side of 3D tri-cultures after Si10 or Min-U-Sil exposure. The figure shows gene expression 3, 6, 10 and 20 h after exposure to 12 and 24 μg/cm^2^ Si10 (**a-d**) or 96 and 192 μg/cm^2^ Min-U-Sil (**e-h**). Gene expression was determined by qPCR, and is presented as fold increase compared to control. Data represent the mean +/− SEM of 3–5 independent experiments. *Statistical significant difference from control at the same time-point *P* < 0.05

Gene expressions of the adhesion molecules ICAM-1, VCAM-1 and E-Selectin were also examined. Exposing the 3D tri-cultures to the lowest concentrations 12 μg/cm^2^ of Si10 increased gene expression of ICAM-1 and E-Selectin on the apical side with significant increases from 6 to 20 h (Fig. 4a, c). At this concentration no increased gene expressions were observed for neither ICAM-1 nor E-selectin on the basolateral endothelial side (Fig. 4b, d). However, upon increasing the exposure concentration to 24 μg/cm^2^ of Si10, gene expression of ICAM-1 was increased by 20-fold in both compartments and for E-Selectin by 400- and 300-fold on the apical and basolateral side, respectively, when measuring at 20 h (Fig. 4a-d). Exposure to Min-U-Sil induced similar effects, with only the highest concentration giving gene expression on the basolateral side. In contrast to Si10, increases in gene-expression reached maximal values already at 3–6 h. Gene expression increase after exposure to 192 μg/cm^2^ was 15- and 30-fold for ICAM-1 on the apical side and basolateral side, respectively, after 6 h exposure. For E-Selectin, gene expressions were approximately similar on both sides (500-fold), when measured after 6 h (Fig. 4g-h). Si10 and Min-U-Sil also induced an increased expression of VCAM-1 on the apical side. However, V-CAM-1 was not expressed on the basolateral side, even in the presence of high concentrations of Si10 and Min-U-Sil (data not shown).

#### Oxidative stress

**Fig. 5:**
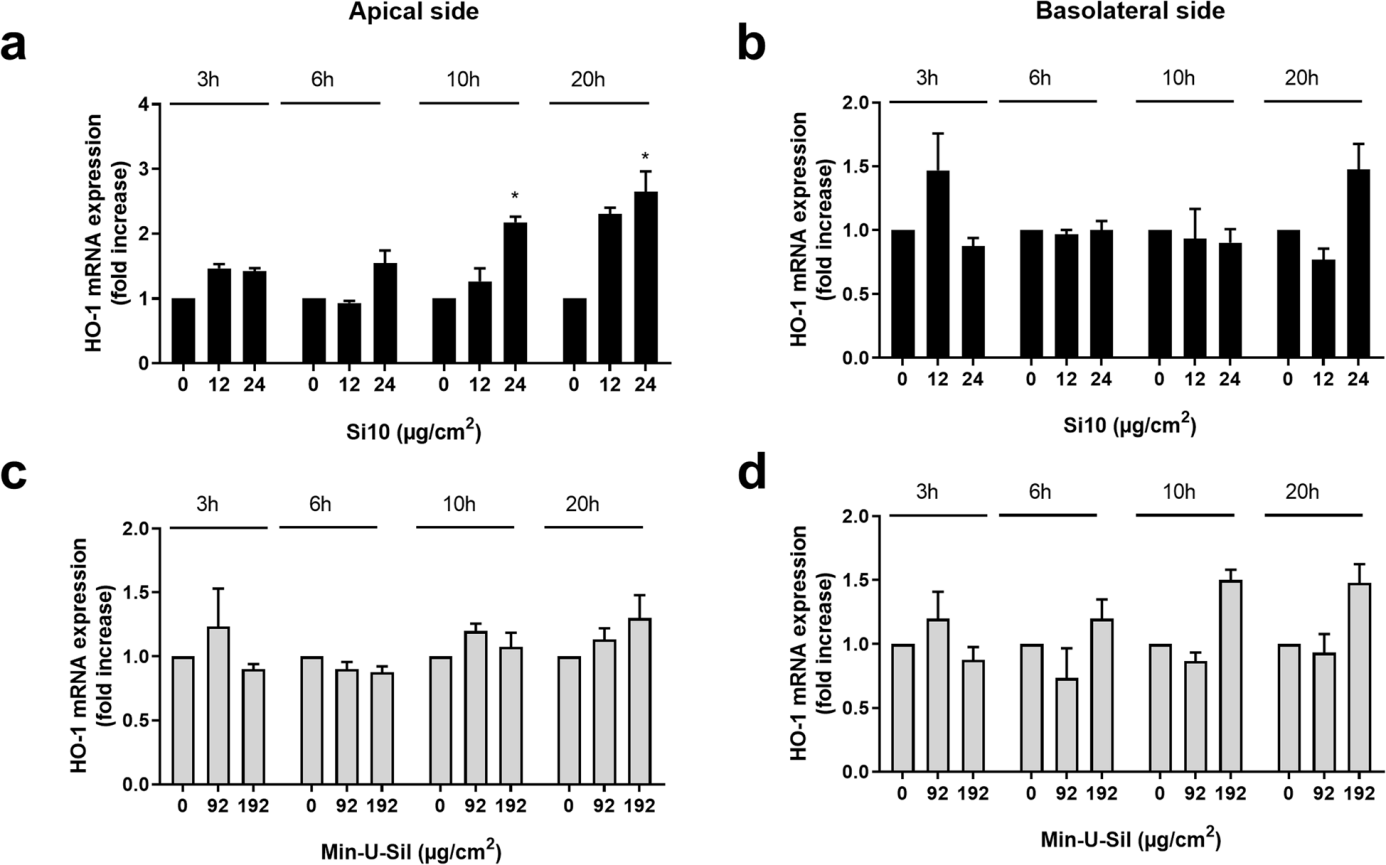
Time-dependent gene expression of HO-1 on the apical and basolateral side of 3D tri-cultures after Si10 or Min-U-Sil exposure*.* The figure shows gene expression after 3, 6, 10 and 20 h exposure to 12 and 24 μg/cm^2^ Si10 (**a**, **b**) and 96 and 192 μg/cm^2^ Min-U-Sil (**c**, **d**). Gene expression was determined by qPCR and is presented as fold increase compared to control. Data represent the mean +/− SEM of 3–5 independent experiments. *Statistical significant difference from control at the same time-point, *P* < 0.05

Gene expression of HO-1, indicative of oxidative stress, was analysed in the 3D tri-culture. The highest exposure of Si10 (24 μg/cm^2^) induced 2- and 2.5-fold increases of HO-1 mRNA after 10 and 20 h (Fig. 5a). This was only observed on the apical side. No significant increases of HO-1 mRNA were observed after exposure to Min-U-Sil, even at the highest concentration (192 μg/cm^2^) (Fig. 5c, d).

#### Metalloproteases

**Fig. 6:**
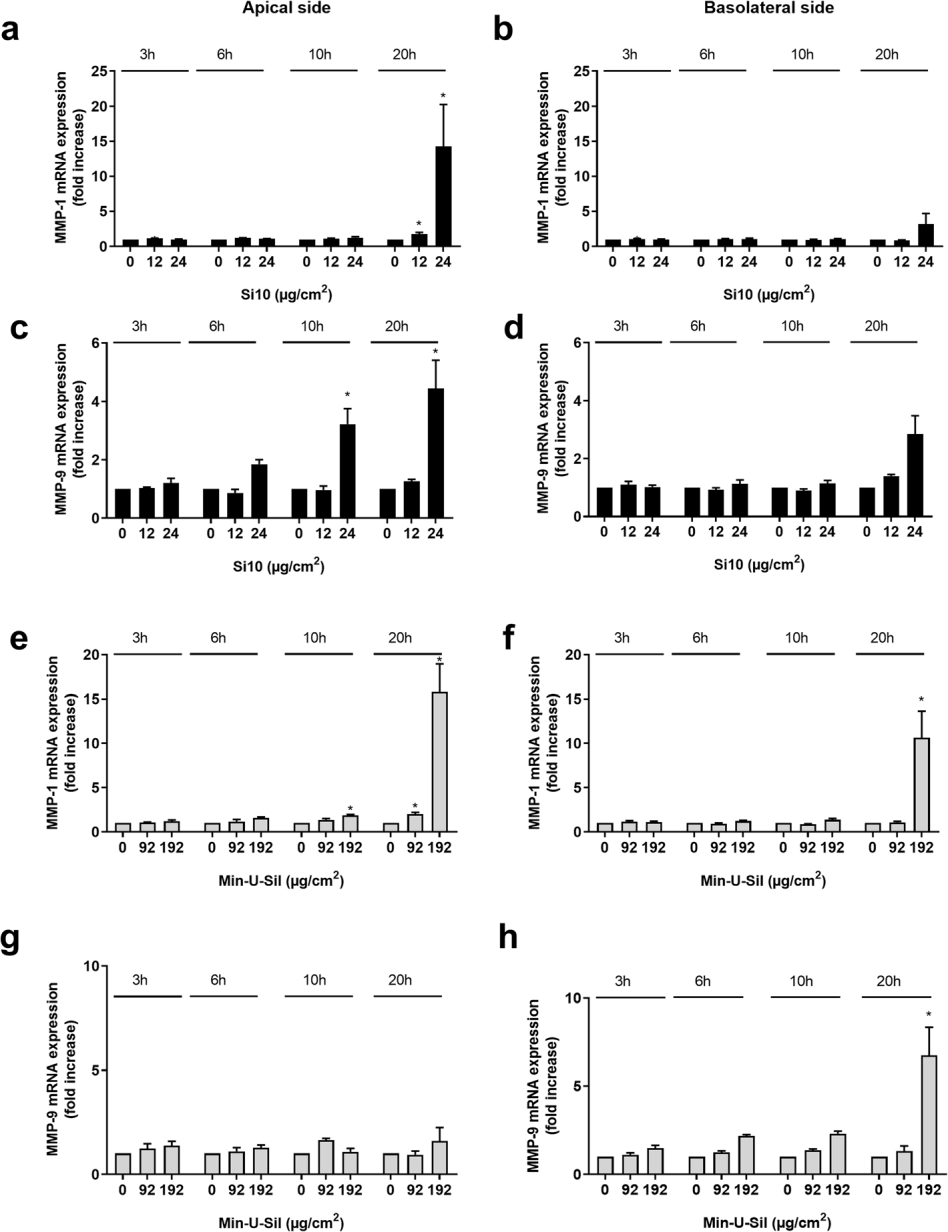
Time-dependent gene expression of MMP-1 and MMP-9 on the apical and basolateral side of 3D tri-cultures after Si10 or Min-U-Sil exposure. The figure shows gene expression after 3, 6, 10 and 20 h exposure to 12 and 24 μg/cm^2^ Si10 (**a**-**d**) and 96 and 192 μg/cm^2^ Min-U-Sil (**e**-**h**). Gene expression was determined by qPCR and presented as fold increase compared to control. Data represent the mean +/− SEM of 3–5 independent experiments. *Statistical significantly difference from control at the same time-point, *P* < 0.05

Gene expressions of the metalloproteases MMP-1 and MMP-9 were also examined in the 3D tri-culture. The highest concentration of Si10 (24 μg/cm^2^) induced a 15-fold increase of MMP-1 after 20 h exposure, and 3- and 4-fold increase of MMP-9 after 10 and 20 h exposure in the apical compartment (Fig. 6a, c). No significant increases were observed in the basolateral compartment (Fig. 6b, d). The highest concentration of Min-U-Sil (192 μg/cm^2^) increased gene expression of MMP-1 in both the apical and basolateral compartment 15- and 10-fold, respectively, after 20 h (Fig. 6e, f). Expression of MMP-9 was only increased on the basolateral side (6.8- fold) after 20 h exposure (Fig. 6g, h).

### Effects of IL-1 receptor antagonist

**Fig. 7:**
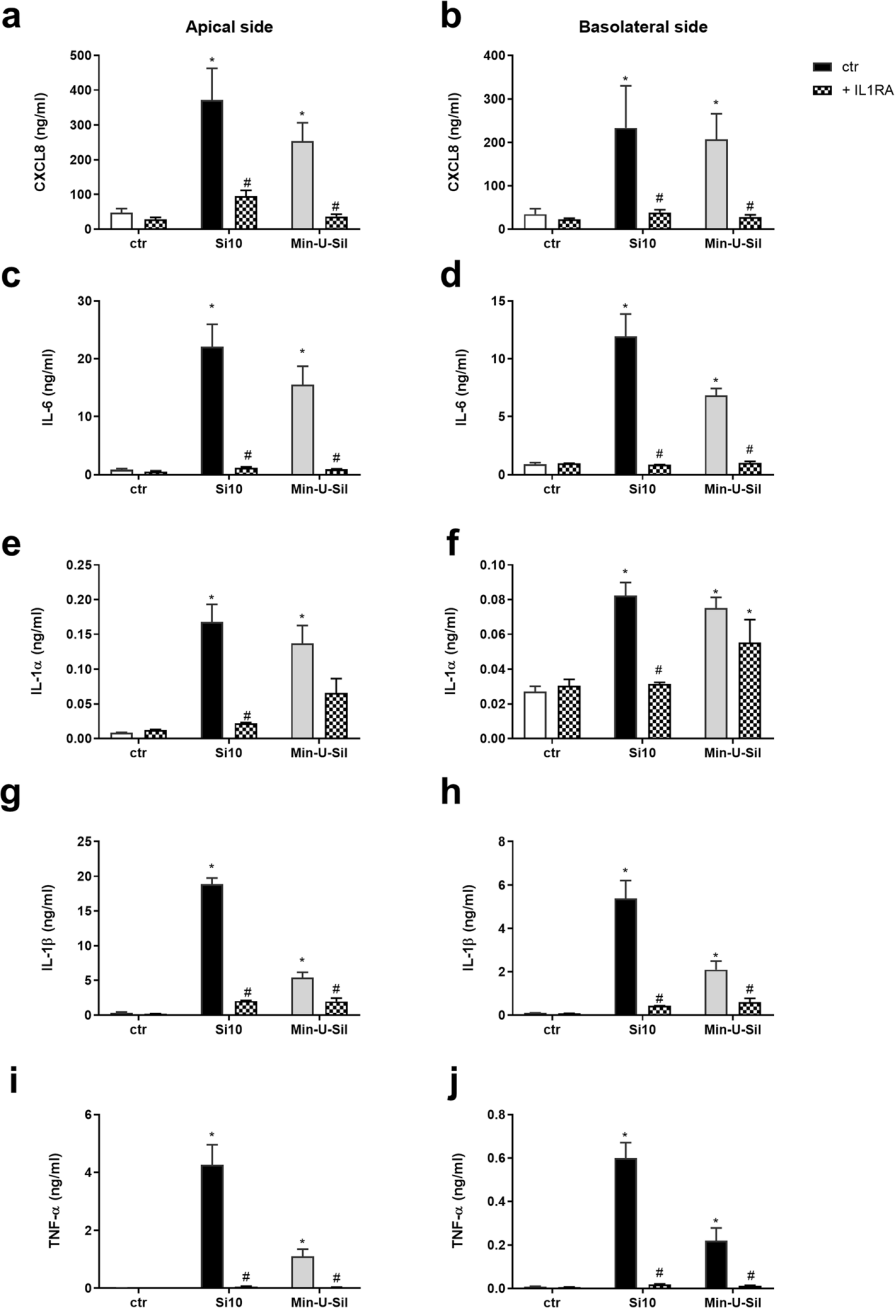
Effect of IL-1RA on CXCL8, IL-6, IL-1α, IL-1β and TNF-α release after exposure to Si10 or Min-U-Sil in 3D tri-cultures. The figure shows cytokine-release on the apical versus the basolateral side of the 3D tri-culture after 20 h exposure. Cells in the apical compartment were pre-treated for 1 h with and without interleukin-1 receptor antagonist (IL-IRA; 30 μg/ml), and exposed for 20 h with and without Si10 (24 μg/cm^2^) or Min-U-Sil (192 μg/ cm^2^) in the presence of the antagonist. Cytokine levels are determined by ELISA, and data represent the mean +/− SEM of 3 independent experiments. *Statistical significantly difference from control, #statistical significant difference from exposure without IL-1RA, *P* < 0.05

**Fig. 8:**
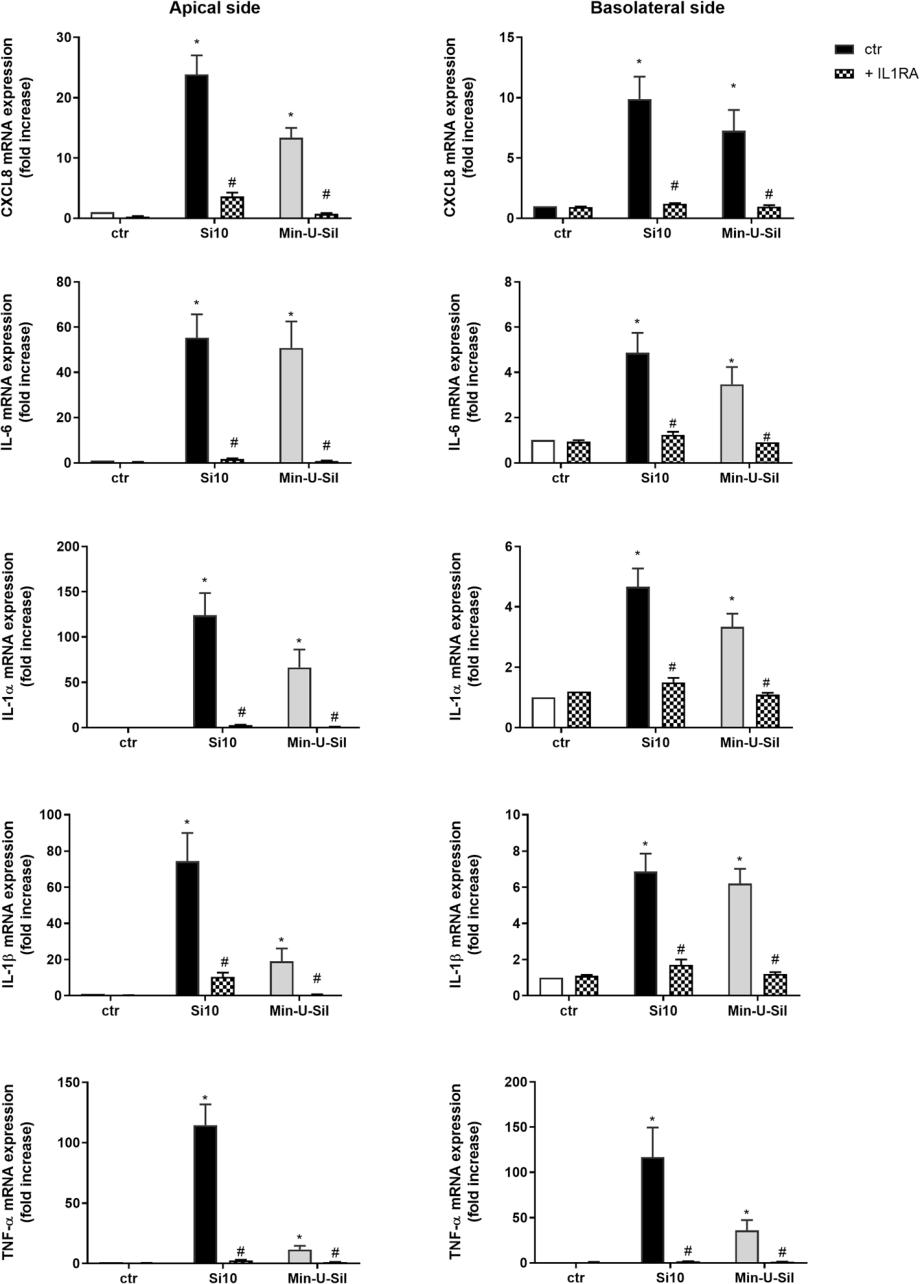
Effect of IL-1RA on CXCL8, IL-6, IL-1α, IL-1β and TNF-α gene expression after exposure to Si10 or Min-U-Sil in 3D tri-cultures*.* The figure shows gene expression on the apical versus the basolateral side of the 3D tri-culture. Cells in the apical compartment were pre-treated for 1 h with and without interleukin-1 receptor antagonist (IL-IRA; 30 μg/ml), and exposed for 10 h with and without Si10 (24 μg/cm^2^) or Min-U-Sil (192 μg/ cm^2.^) in the presence of the antagonist. Gene expression was determined by qPCR and represent the mean +/− of 3 independent experiments. *Statistical significant difference from control, #statistically significantly different from exposure without IL-1RA, *P* < 0.05

**Fig. 9:**
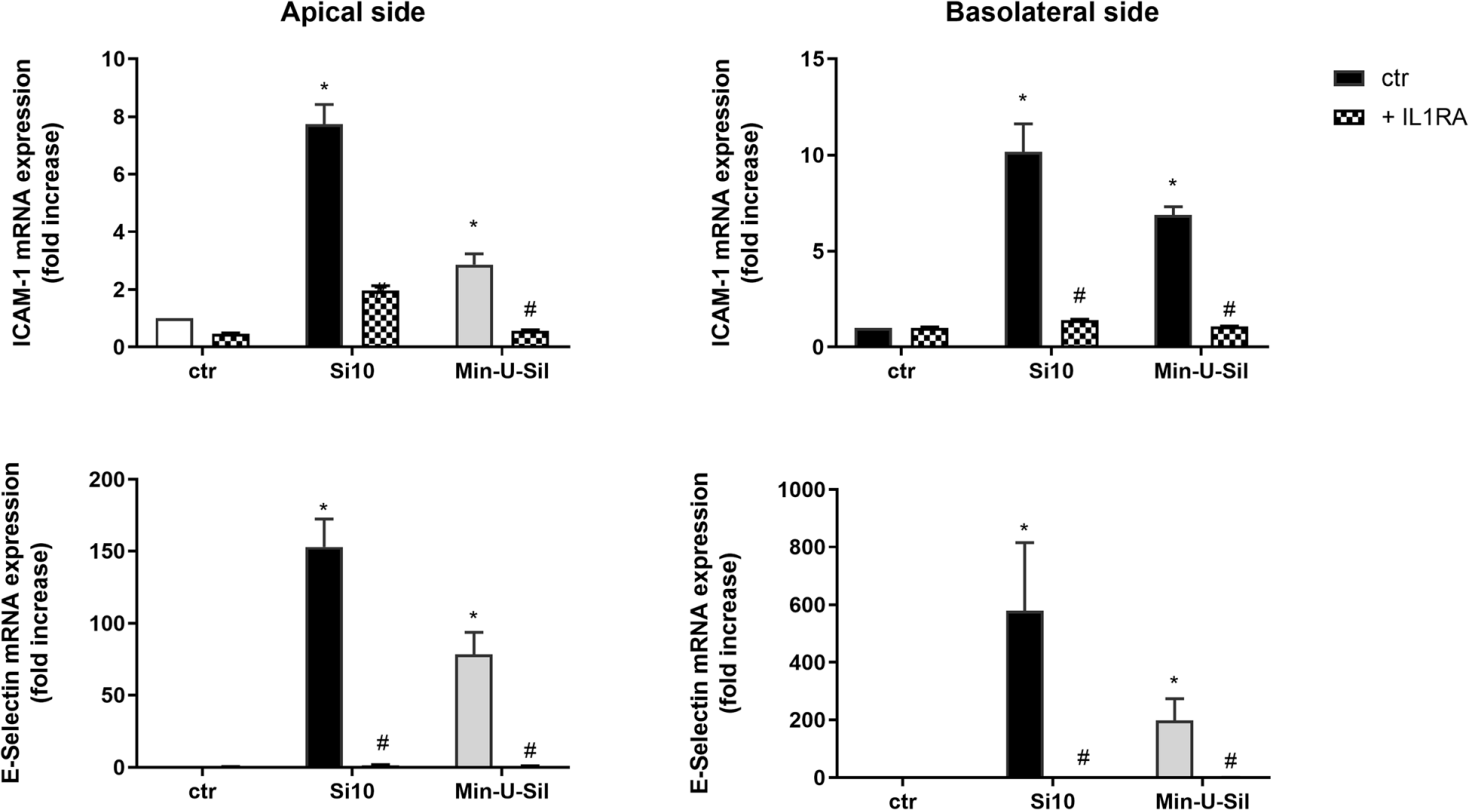
Effects of IL-1RA on ICAM-1 and E-Selectin gene expression after exposure to Si10 or Min-U-Sil in 3D tri-cultures*.* The figure shows gene-expression on the apical versus the basolateral side of the 3D tri-culture. Cells in the apical compartment were pre-treated for 1 h with and without interleukin-1 receptor antagonist (IL-IRA; 30 μg/ml), and exposed for 10 h with and without Si10 (24 μg/cm^2^) or Min-U-Sil (192 μg/ cm^2.^) in the presence of the antagonist. The gene expression was determined by qPCR and represent the mean +/− of 3 independent experiments. *Statistical significant difference from control, #statistical significant difference from exposure without IL-1RA, *P* < 0.05

To examine the potential role of IL-1α and IL-1β in the pro-inflammatory responses in the 3D tri-cultures, the IL-1R antagonist IL-1RA (30 μg/mL) was added to the apical compartment 1 h before exposure to Si10 (24 μg/cm^2^) and Min-U-Sil (192 μg/cm^2^). The releases of pro-inflammatory cytokines and the gene expressions were examined at 20 and 10 h, respectively (Figs. 7 and 8). IL-1RA almost completely blocked release of CXCL8, IL-6, IL-1β and TNF-α after exposure to the high concentrations of both Si10 and Min-U-Sil, whereas IL-1α was less reduced, in particular after Min-U-Sil exposure (Fig. 7). Statistically significant reduced cytokine release was observed in both the apical and basolateral compartments. Effects of both Si10 and Min-U-Sil on gene expressions of all the pro-inflammatory cytokines (10 h exposure) were also almost completely blocked on both sides of the co-cultures (Fig. 8). Notably, the same results appeared if IL-1RA was added in both chambers or only on the apical side (data not shown), indicating that IL-1RA diffuses between the two chambers, and inhibits gene expression and cytokine release completely. ICAM-1 and Selectin-E gene expressions were also almost completely inhibited by IL-1RA (Fig. 9).

## Discussion

Using a 3D tri-culture lung model, we found that non-crystalline SiNPs (Si10) and crystalline silica particles of micro-meter size (Min-U-Sil) induced gene expression and release of pro-inflammatory cytokines in the apical epithelial/macrophage compartment and the basolateral endothelial compartment. Effects were time- and concentration-dependent. Endothelial gene expression were mainly exerted at higher concentrations, delayed and of less magnitude when compared to the apical macrophage/epithelial cell compartment. Furthermore, Si10 seemed more potent than Min-U-Sil on a mass basis, but effects of Si10 were delayed. Similar findings were observed for gene expression of adhesion molecules. An IL-1R antagonist almost completely blocked silica particle-induced up-regulation of cytokine release, and cytokine and adhesion molecule gene expression in both compartments. Thus, the present study suggests that IL-1α/β are central mediators of pro-inflammatory responses in both compartments of the 3D tri-culture.

The findings with transfer of pro-inflammatory mediators across the cell barrier expand the knowledge of our previous studies with Min-U-Sil using a 2D co-culture system involving THP-1 monocytes, A549 cells and another endothelial cell line (HIVE-26) [50]. Effects at lower concentrations and the higher magnitude of endothelial responses seen in the present study may be due to the 3D organization, which brings the endothelial cells in closer contact with the A549 cells and/or the addition of THP-1 macrophages. Use of THP-1 macrophages has been shown to have a large impact on the complexity of the system [44, 48, 59]. We previously observed that also non-differentiated THP-1 monocytes exposed to Min-U-Sil in the apical compartment (in absence of A549 cells) increased the gene expression of cytokines in endothelial cells in the basolateral compartment [49]. Thus, apparently both addition of THP-1 monocytes and macrophages will result in co-culture models with increased cytokine responses.

Our present data, show that addition of an IL-R antagonist almost completely blocked effects of Min-U-Sil and Si10 on gene expression of cytokines and adhesion molecules in the apical epithelial cells and macrophages, as well as endothelial cells on the basolateral side. As increased IL-1α/β expression is required for an up-regulation of the gene expression of cytokines and adhesion molecules in both cell compartments, the data suggest that IL-1α/β cytokines from the apical side cross over the cell barrier to the basolateral endothelial cells. Notably, we have previously shown that IL-1β is released from mouse macrophage (RAW264.7) upon exposure to different types and sizes of silica particles [12]. IL-1α is an alarmin, known to be released from both macrophages [60] as well as epithelial cells upon exposure to various agents [61, 62]. Also IL-1β is released from both macrophages and epithelial lung cells upon exposure to silica particles [12, 18]. IL-1β release is due to silica particle-induced activation of the inflammasome due to uptake of particles causing destabilization of the lysosomal membrane [12, 16, 18, 63, 64]. Furthermore, we have in previous studies found that SiNPs also are able to induce gene expression and release of IL-1α and IL-1β in lung epithelial cells at rather low concentrations [11]. Interestingly, IL-1α and IL-1β are both known to be critical paracrine (and endocrine) mediators of inflammation linked to lung [34, 60, 65, 66] and cardiovascular disease [35, 36]. A number of in vitro studies have also illustrated that these cytokines may trigger other inflammatory responses. We have previously reported that an IL-R antagonist reduced Min-U-Sil-induced cytokine release in different co-culture systems, involving THP-1 monocytes, alveolar lung epithelial cells (A549) and endothelial cells (HIVE 26), suggesting that IL-1 is important for the interplay between the different cell types [49, 50, 67]. In the present study we show in a more complex 3D co-culture system that cytokine and adhesion molecule responses are due to IL-1-dependent gene expression.

In our 3D tri-culture, concentrations of IL-1 in the apical compartment was higher than in the basolateral compartment, suggesting that the IL-1 signalling may start on the apical side. This may also explain why enhanced gene expression of cytokines and adhesion molecules are seen at lower concentrations of silica particles in the apical macrophages/epithelial cells than compared to basolateral endothelial cells. Similarly, the up-regulations in endothelial cells are lower in magnitude and time-delayed compared to macrophages/epithelial cells. This may be related to time-points and concentrations at which sufficient IL-1 levels are reached in basolateral compartment to trigger effects. These findings presumably reflect what occurs in vivo upon inhalation exposure of particles, with systemic responses of much less magnitude and appearing at later time-points than in the airways. This suggests that such 3D co-culture systems may have properties that make them suitable to examine some aspects of particle toxicology, and might to some extent replace in vivo studies.

The IL-1R antagonist used in this work is not specific, as its affects both IL-1α and IL-1β ligand binding. Since release of IL-1β strongly exceeds the amounts of IL-1α, this may suggest that IL-1β is the critical signalling molecule. This is supported by the fact that while IL-1β appears to have a controlled release at non-cytotoxic concentrations, release of IL-1α is more often parallel to cell death. However, IL-1α has also been reported to be an alarmin and a master cytokine that induces acute lung inflammation via pro-IL-1β synthesis and IL-1β release [60]. Furthermore, as the responses will depend on the affinity of IL-1α and IL-1β towards the IL-1 receptor, we will not draw any final conclusion on the relative role of these IL-1R ligands. Further studies are required to elucidate this. Overall, our present findings support that IL-1α/β act as paracrine regulators, promoting interaction between THP-1 macrophages and A549 cells. THP-1 cells have also been reported to activate the endothelial cells separated from the macrophages/lung epithelial cells by a micro-porous membrane (pore size 1 μm), which these cells cannot penetrate [44]. It is interesting to note that we have demonstrated a similar IL-1-dependent regulation of pro-inflammatory responses in co-cultures of primary cardiac fibroblasts and cardiomyocytes [68], indicating IL-1 to be a general mediator in paracrine interactions of different cell types. Although, the present evidence supports a paracrine role of IL-1, responsible for the interplay between THP-1 macrophages, A549 cells and endothelial cells, IL-1 may also have a potential role to act at longer distances from the releasing cell source. Accordingly, in our previous tri-cultures which also suggested a role for IL-1, the endothelial cells were localized at the bottom of the wells [49]. Furthermore, we have shown that transfer of conditioned medium from primary rat lung epithelial type 2 cells exposed to ultrafine Printex particles enhanced release of IL-6 and CXCL8 from rat primary cardiac co-cultures in a partial IL-1-dependent manner [68]. Thus, IL-1 released due to airway exposure to Min-U-Sil- and Si10 may potentially cause systemic effects. This will obviously depend on the concentration of IL-1 reaching different target organs.

In our 3D co-culture system rather high concentrations of silica particles are required for eliciting sufficient high concentrations of IL-1 to trigger endothelial cytokine gene expression. A critical question is whether sufficient high concentrations of IL-1 will be reached in plasma during in vivo silica particle exposure. Previously, it has been reported that amorphous silica particles (Si50, Si500) upon intra-tracheal administration (50 mg/kg) in mice increased plasma IL-β concentrations to 0.2–0.3 ng/ml [69]. Such concentrations would not induce an endothelial gene expression in our co-culture system, however it should emphasized that such extrapolation from in vivo to in vitro settings, are difficult and should be carefully interpreted. The concentrations of silica particles used in the present study are in the concentrations range of previous in vitro studies [1, 70]. When using the estimation method of Li and coworkers 2003 [71], the concentrations of Min-U-Sil (24–192 μg/cm^2^) in our co-cultures (apically) seem to be in the upper range or somewhat above what should be expected from occupational exposure, with observed silica concentrations up to 0.6 mg/m^3^ and an EU occupational exposure limit (OEL) of 0.1 mg/m^3^. For SiNPs the knowledge of exposure levels are lacking [72], making it difficult to estimate the relevant concentrations for in vitro exposure.

One other major hypothesis for systemic effects of NPs, is that NPs may translocate from the airways into the circulation, thus reaching secondary organs, and triggering inflammation and pathogenic processes. Accordingly, gold-NPs have been reported at atherosclerotic plaques, however only small amounts of inhaled NPs seem to translocate into the blood [39]. The critical question is whether these concentrations are sufficient to trigger inflammatory responses [39, 40]. Although not specifically addressed in the present in vitro study, a question is whether SiNPs may pass the epithelial barrier and the micro-porous membrane in 3D tri-cultures, and thereby exert direct effects on endothelial cells. Rhodamine-labelled Si50 added to the presently used 3D tri-culture was only located in THP-1 macrophages and not in A549 cells nor endothelial cells [44]. In contrast, translocation of NPs across the filter/membrane barriers to endothelial cells has been reported in a rather similar 3D co-culture model [73]. This may depend on the particle size and its agglomeration state as well as the pore size of the filter. In the current study we used filters with pore size of 1 μm, while SiNPs had a lower nominal size of 10 nm, and will penetrate the filter without cells. Thus we cannot exclude that Si10 could pass across the cell barrier, but we presume that this does not occur in sufficient amounts to trigger a response in the endothelial cells. In support of this, the passage of sodium fluorescein across the cell barrier in the 3D co-cultures was rather low. Furthermore, Min-U-Sil particles with a nominal median size of 1.4 μm that could not pass the filters, still caused very similar responses as Si10. Finally, upon assuming that a high amount of Si10 particles could pass the cell barrier on the insert membrane, we examined the potential of Si10 to directly activate the endothelial cells in mono-cultures. These studies showed that rather high concentrations of Si10 were required to induce cytokine responses, and most importantly, the cytokine response pattern in the mono-cultures was qualitatively different from the pattern in the 3D co-cultures; with no IL-1β and TNF-α responses in the monocultures. Overall this suggests that IL-1, and not Si10 pass the cell barrier, and is the critical determinant in eliciting endothelial responses in our co-culture model.

Our data show that Si10 was more potent than Min-U-Sil on the gene expression in both compartments when related to PM mass, whereas Min-U-Sil was more potent when related to PM surface area. (Fig. S3). The experiments were performed by using submerged culture conditions and different dispersion conditions. Conceivably, using ALI-exposure and similar dispersants could have influenced the relative potency. Furthermore, different mechanisms for uptake of the silica particles of different sizes would affect the potency for induction of pro-inflammatory responses and cytotoxicity [8–10]. The early up-regulation of genes in both compartments seen after exposure to Min-U-Sil compared to Si10 particles in the submerged co-culture is presumably due to the higher sedimentation rate and faster contact with the apical cells.

A role for both ROS and metalloprotease activity has been reported for particle- and NP-induced cytokine gene expression [17, 74, 75]. Here enhanced gene expressions of HO-1, MMP-1 and MMP-9 were only seen at later time-points. This suggests that the induced gene expression of HO-1 and metalloproteases are not necessary for the acute Si10- and Min-U-Sil-induced changes seen in pro-inflammatory genes. Up-regulation of HO-1, MMP-1 and -9 may possibly be related to downstream effects of earlier cytokine responses. Similarly, exposing the same 3D tri-culture to DEP, MMP-1 was only induced at the latest time point [53].

It is important to emphasize that our 3D co-culture is only a model, and that it needs to be further improved. Results from such models cannot be directly extrapolated to in vivo settings. A low trans-epithelial transport of sodium fluorescein across the cell barrier was observed in our model, indicating a relatively tight cell barrier. On the other hand, the trans-epithelial resistance (TEER)-value was rather low, indicating that A549 cells have no functional tight junctions [44]. Theoretically, IL-1α/β may be actively transported from the apical epithelial cells/macrophages across the alveolar barrier to the basolateral endothelial cells, or passively diffuse across the barrier via non-functional cellular tight junctions. Several studies have also shown that exposure to toxicants may reduce TEER values [73]. This may result in unspecific leakage of cytokines across the barrier. Presumably, this is not occurring in the present study, as neither Si10 nor Min-U-Sil induced any toxicity. However, to more properly address the importance of IL-1 in 3D co-culture systems, there is a need to optimize the models to have tight alveolar-endothelial cell barriers. To achieve a more functional lung cell barrier other epithelial lung cell lines need to be introduced to replace A549 cells, to better mimic the in vivo situation [73, 76–79]. As A549 cells also are known to be less responsive to different toxicants [80] than many other lung epithelial cell lines, and in particular compared to primary type 2 cells, replacement of A549 cells with other lung epithelial cell types, might enhance the sensitivity of the model system. The ratio between THP-1 cells and A549 cells in our model is approximately 1 to 2.5, which is in the upper range of what could be expected for the ratio between macrophages and type 2 epithelial lung cells in alveoli from humans [81]. Thus, the number of macrophages in our co-culture model seems rather high, making it most representative for lung-disease states. Notably, it has been reported that the macrophage number in the human alveoli is approximately 100/ mm^2^ and 200/ mm^2^ in healthy, non-smoking individuals and COPD-patients, respectively [82]. Presently, work is in progress to introduce other epithelial lung cell lines, and to examine the influence of the macrophage number in the co-culture model, to improve and make the models as representative as possible for in vivo conditions.

## Conclusions

Silica nanoparticles (Si10) and crystalline silica (Min-U-Sil) induced pro-inflammatory responses in a concentration- and time-dependent manner in both compartments of our 3D tri-culture, containing lung epithelial cells and macrophages in the apical compartment and endothelial cells in the basolateral compartment. Gene expression in the basolateral endothelial cells was only triggered at high concentrations of the silica particles and was delayed compared to responses in the apical compartment. Furthermore, the responses in both compartments seemed to be triggered by release of IL-1α and/or IL-1β.

## Methods

### Materials

The cell culture media DMEM (1X), Glutamax and RPMI Medium 1640 (1x) and Glutamax and Trypsin-EDTA were bought from Gibco, Thermo Fisher Sientific, Waltham, MA, USA. Fetal bovine serum (FBS Superior) was obtained from BIOCROM AG, Berlin, Germany. The cell culture flasks were obtained from Nunc A/S, Roskilde, Denmark, while the cell culture inserts (1 μm pores) and cell culture plates were from Falcon, Corning, NY 14831 USA. Phorbol-12-myristate-13-acetate (PMA) and lipopolysaccharides (LPS) were from Merck, Darmstadt, Germany. Accutase (A6964) was bought from Sigma-Aldrich, Merck (Darmstadt, Germany). The ELISA cytosets for IL-6, CXCL8/IL-8 and TNF-α were purchased from Invitrogen, Thermo Fisher Scientific, Waltham, MA, USA, while IL-1α and IL-1β DuoSet were from R&D Systems, Inc., Minneapolis, MN, USA. The RNA isolating kit; RNeasy Mini Kit, DNase set I and QIAshredder were bought from Qiagen (Germantown, MD, USA). Alamar blue, High Capacity cDNA Archive Kit, TaqMan Universal PCR Mastermix, TaqMan Gene Expression Assays (CXCL8, Hs00174103_m1, IL-6, Hs00174131_m1, IL-1α; Hs00174092_m1, IL-1β; Hs01555410_m1, TNF-α; Hs01113624_g1, ICAM-1 (Hs00164932_m1), VCAM-1 (Hs01003372_m1), E-Selectin (Hs00174057_m1), MMP-1 (Hs00899658_m1), MMP-9 (Hs00957562_m1), HO-1 (Hs01110250_m1), GAPDH; Hs02758991_g1 and HPRT1; Hs02800695_m1 were bought from Applied Biosystems, Thermo Fisher Scientific, Waltham, MA, USA. Anakinra (IL1RA) was obtained from Kinret, Amgen, BV, Breda, Netherlands. Pierce Chromogen Endotoxin Quant kit was from Thermo Fisher Scientific, Waltham, MA, USA. Other chemical were purchased from commercial sources at the highest purity available.

### Particles

The silica NPs with a nominal size of 10 nm (Si10) were obtained from Kisker Biotech (Steinfurt, Germany) and Min-U-Sil®5 ground silica (quartz) was given to us from U.S. Silica Company, Berkeley Springs, WV, USA.

### Si10 and Min-U-Sil solutions, dissolvents and characterization

The silica nanoparticle solution sized 10 nm (Si10), was diluted in PBS with 0.15% bovine serum albumin (BSA) after a sonication step (420 J) according to the method of Bihari et al.2008 [11, 83]*.* Min-U-Sil was suspended in co-culture media (2 mg/mL) and sonicated in the same manner as Si10. Characterisation of Si10 was done in a previous study, showing a particle size of 10.9 nm, a surface area of 243.6 m^2^/g and a zeta potential of 41.6 mV [11]. According to the manufacturer, the size of Min-U-Sil particles has a median size of 1.6 μm with 96% less than 5 μm, and containing up to 99.2% crystalline silica, with a surface area of 7.2 m^2^/g. Dynamic light scattering (DLS) analyses of Si10 and Min-U-Sil in co-culture media were performed in a zeta-sizer nano (Malvern Inc., Germany) and revealed a size of 166 nm for Si10 and 1600 nm for Min-U-Sil. For both particle-solutions the polydispersity index (PdI value) indicated a one-particle population. The endotoxin content was measured by the Pierce Chromogen Endotoxin Quant kit (LAL-method), and showed 0.01 EU/ml for Min-U-Sil and 0.082 EU/ml for Si10. LPS added to the 3D co-cultures in concentrations equivalent to concentrations of endotoxin in Si10 was without effect on cytokine release (Fig. S4).

### Culturing of cells; building the 3D tri-culture and exposure

The 3D tri-culture model, established as described by Klein and co-workers [43, 44], consisted of three different cell-types, A549 epithelial lung cells, PMA-differentiated THP-1 cells, and EA.hy 926 endothelial cells (from the American Type Culture Collection, Manassas, VA, USA), and has been used by us in a previous study [53]. The Ea.hy 926 cells were maintained in DMEM + Glutamax, with HEPES buffer (DMEM) and 10% FBS. A549 cells were maintained in DMEM + Glutamax and 10% FBS, and the THP-1 cells were maintained in RPMI-1640 with Glutamax and 10% FBS. All the cells were cultured in T75 flasks in a humidified atmosphere at 37 °C with 5% CO_2_, with refreshment of medium twice a week. Building the 3D co-cultures started by seeding EA.hy 926 cells on the inverted trans-well inserts with 4.2 cm^2^ surface area and 1 μm pores at a density of 2.6 × 10^5^ cells/cm^2^. Four hours after seeding, the trans-well inserts were turned back to its original orientation and A549 cells were seeded inside the trans-well (1.28 × 10^5^ cells/cm^2^). Epithelial and endothelial cells were then grown for 3 days at 37 °C and 5% in a humidified incubator with 2.0 mL of DMEM Glutamax with 10% FBS in the apical (upper) and the basolateral (lower) chamber. The medium was changed to 2 mL co-culture media (DMEM Glutamax with 15% RPMI-1640 and 10% FBS) in both chambers 3 days later, and the THP-1 monocytes in monocultures were differentiated into macrophage-like cells (THP-1 macrophages) with PMA (20 ng/mL) for 24 h. The THP-1 macrophages were then detached using Accutase solution (5 mL) for 15 min and added to inserts at a concentration of 2.6 × 10^5^ cells/cm^2^. The complete 3D tri-culture was kept in co-culture media with 1% FBS one more day before the 3D tri-culture was ready for exposure. The cell numbers before exposure were estimated, based on number of cells seeded, attachment and cell proliferation, to be approximately 2 million A549 cells, 0.8 million THP-1 cells (the attachment was approximately 75%) and 2 million Ea.hy 926. The permeability of the cell barrier in the 3D co-cultures was measured by the penetration of Sodium Fluorescein (10 μg/ml in media) from the apical side to the basolateral side after 1 h. Approximately 8% penetrated across the membrane, suggesting that the cell barrier was relatively tight, but did not completely prevent passage of these molecules.

In the experiments the cells were exposed to Si10 (0–100 μg/mL, corresponding to 0–48 μg/cm^2^) and Min-U-Sil (0–400 μg/mL, corresponding to 0–192 μg/cm^2^) for 2, 6, 10 and 20 h under submerged conditions in co-culture media without FBS. In some experiments the cells were pre-treated for 1 h and treated during the exposure period with interleukin-1 receptor antagonist (IL-1RA; 30 μg/mL).

### Exposure of endothelial cells in mono-cultures to Si10

Ea.hy 926 cells in mono-cultures were exposed to Si10 (0–100 μg/mL, corresponding to 0–48 μg/cm^2^) in DMEM Glutamax for 20 h; and analysed for cytokine release, and viability as described below.

### Cell viability

The viability was assessed by measuring metabolic activity after 20 h of exposure, using an Alamar Blue kit and performed as described in the producer’s manual. The signal was measured by fluorescence on a fluorimeter (CLARIO STAR).

### Cytokine analysis

Cell culture media from the inserts (apical side) as well as from the cell culture well (basolateral side) were collected and centrifuged at 300 x g to remove cell debris and at 8000 x g to remove floating silica particles. The protein levels of CXCL8, IL-6, IL-1α, IL-1β and TNF-α were determined by sandwich ELISA according to the manufacturer’s guidelines. Absorbance was measured and quantified by a plate reader (TECAN Sunrise) equipped with dedicated software (Magellan V 1.10).

### RNA isolation and gene expression analysis (QPCR)

After exposure to Si10 and Min-U-Sil, cells were washed twice with PBS, before cells on the endothelial side were lysed by scraping with a cotton pad wet with lysis buffer. After collecting the endothelial celllysate, cells in the apical compartment were harvested by adding lysis buffer into the well. Cellular RNA was isolated according to the supplier’s recommendations, using QIAshredder colons and RNeasy Mini Kit. RNA was treated with DNase I to ensure RNA without DNA. RNA concentrations were determined on a spectrophotometer (DS-11, DeNovix (Wilmington, DE 19810 USA)). Before analysing gene expression (IL-6, CXCL8, IL-1α, IL-1β, TNF-α, ICAM-1, VCAM-1, E-Selectin, MMP-1, MMP-9 and HO-1) with qPCR, 1 μg total RNA was reverse transcribed to cDNA using a High Capacity cDNA Archive Kit. The cDNA was further diluted in the qPCR reaction 1:1000. The QPCR reaction was done on Applied Biosystems 7500 Real-Time PCR System or on BioRads CFX96 Touch Real-Time PCR Detection System with pre-designed TaqMan Gene Expression Assays. The expression of each gene of interest (GOI) in each sample was normalized against two housekeeping genes (GAPDH, HPRT1) and expressed as fold change compared to untreated controls, calculated by the ΔΔCt method (ΔCt = Ct [Gene of Interest] – Ct [GAPDH/HPRT1]; ΔΔCt = ΔCt [Treated] – ΔCt [Control]; Fold change = 2[−ΔΔCt]).

### Statistical analyses

Statistical analyses on cytokine release of 5 independent biological replicates were performed by using two-way ANOVA with Dunnett’s post-test for multiple comparisons test. Statistical calculations for the Real time PCR experiments (4 experiments) were done on the dCt-values by two-way ANOVA with Dunnett’s post-test for multiple comparisons. GraphPad Prism software (version 7.0 Inc., San Diego, CA) was used for the analyses.

## Supplementary information

**Additional file 1: Figure S1.** Concentration-dependent viability in 3D tri-culture after exposure to Si10 and Min-U-Sil. The 3D tri-culture consisted of THP-1 macrophages and A549 cells in the apical compartment and EA.hy 926 endothelial cells in the basolateral compartment as described in Material and Methods. The figure shows the viability in the apical and the basolateral compartment after 20 h exposure to Si10 (0–48 μg/cm^2^) and Min-U-Sil (0–192 μg/cm^2^). Viability was determined by AlamarBlue, and data represent the mean +/− SEM of 5 independent experiments.

**Additional file 2: Figure S2.** Concentration-dependent release of IL-1α, CXCL8 and IL-6 after Si10 exposure in endothelial cells. The Ea. Hy 926 cells in monocultures were exposed to Si10 (0–48 μg/cm^2^) for 20 h. The cytokine levels were determined by ELISA, and the results are presented as the mean +/− SEM of 3 independent experiments.

**Additional file 3: Figure S3.** Concentration-dependent release of IL-1β after Si10 and Min-U-Sil exposure in 3D tri-culture related to particle mass and surface area. The figure shows cytokine levels in the apical and the basolateral compartment after 20 h exposure to 0–48 μg/cm^2^ Si10 and 0–192 μg/cm^2^ Min-U-Sil equivalent to 0–25 cm^2^/cm^2^ of Si10 and 0–3 cm^2^/cm^2^ of Min-U-Sil. Cytokine levels were determined by ELISA, and data represent the mean +/− SEM of 5 independent experiments.

**Additional file 4: Figure S4.** Effect of LPS on CXCL8 release in the 3D tri-culture. The 3D tri-culture consisted of THP-1 macrophages and A549 cells in the apical compartment and Ea.hy 926 endothelial cells in the basolateral compartment as described in Material and Methods. The figure shows the CXCL8 release in the apical and the basolateral compartment after 20 h exposure to LPS (0.016 and 10 ng/ml LPS)

## Data Availability

The datasets used and/or analysed during the current study are available from the corresponding author on reasonable request.

## Abbreviations

SiNPs: Silica nanoparticles
NPs: Nanoparticles
IL-6: Interleukin-6
TNF-α: Tumour necrosis factor-α
IL-1α: Interleukin-1α
IL-1β: Interleukin-1β
HO-1: Heme oxygenase-1
IL1-RA: Interleukin-1 receptor antagonist
COPD: Chronic obstructive pulmonary disease
AHR: Aryl hydrocarbon receptor
MMP: Metalloproteases
LPS: Lipopolysaccharides
