# Supplementary figures and images for: Pro-inflammatory effects of crystalline- and nano-sized non-crystalline silica particles in a 3D alveolar model
Source: Part Fibre Toxicol. 2020 Apr 21;17:13. doi: 10.1186/s12989-020-00345-3 (PMC7175518; doi:10.1186/s12989-020-00345-3)

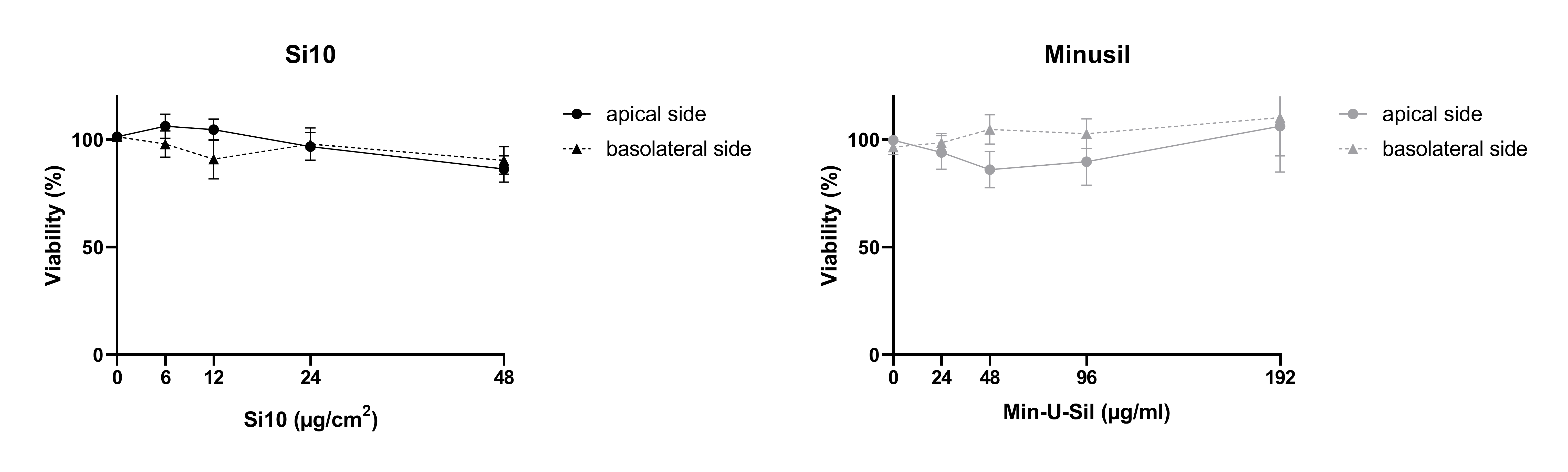

Supplement: Supplementary file 1 — Additional file 1: Figure S1. Concentration-dependent viability in 3D tri-culture after exposure to Si10 and Min-U-Sil. The 3D tri-culture consisted of THP-1 macrophages and A549 cells in the apical compartment and EA.hy 926 endothelial cells in the basolateral compartment as described in Material and Methods. The figure shows the viability in the apical and the basolateral compartment after 20 h exposure to Si10 (0–48 μg/cm2) and Min-U-Sil (0–192 μg/cm2). Viability was determined by AlamarBlue, and data represent the mean +/− SEM of 5 independent experiments. [file 12989_2020_345_MOESM1_ESM.tif]

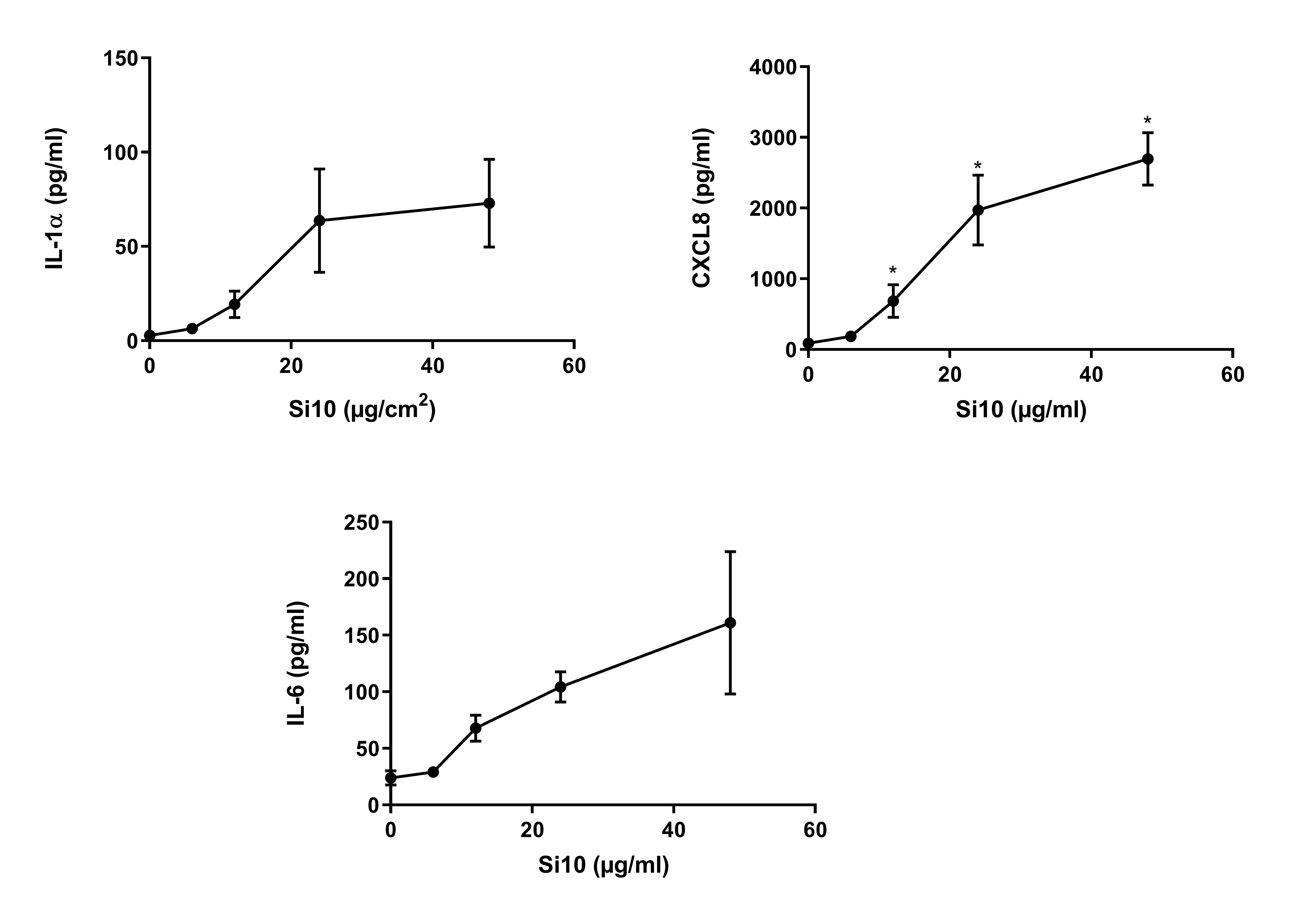

Supplement: Supplementary file 2 — Additional file 2: Figure S2. Concentration-dependent release of IL-1α, CXCL8 and IL-6 after Si10 exposure in endothelial cells. The Ea. Hy 926 cells in monocultures were exposed to Si10 (0–48 μg/cm2) for 20 h. The cytokine levels were determined by ELISA, and the results are presented as the mean +/− SEM of 3 independent experiments. [file 12989_2020_345_MOESM2_ESM.tif]

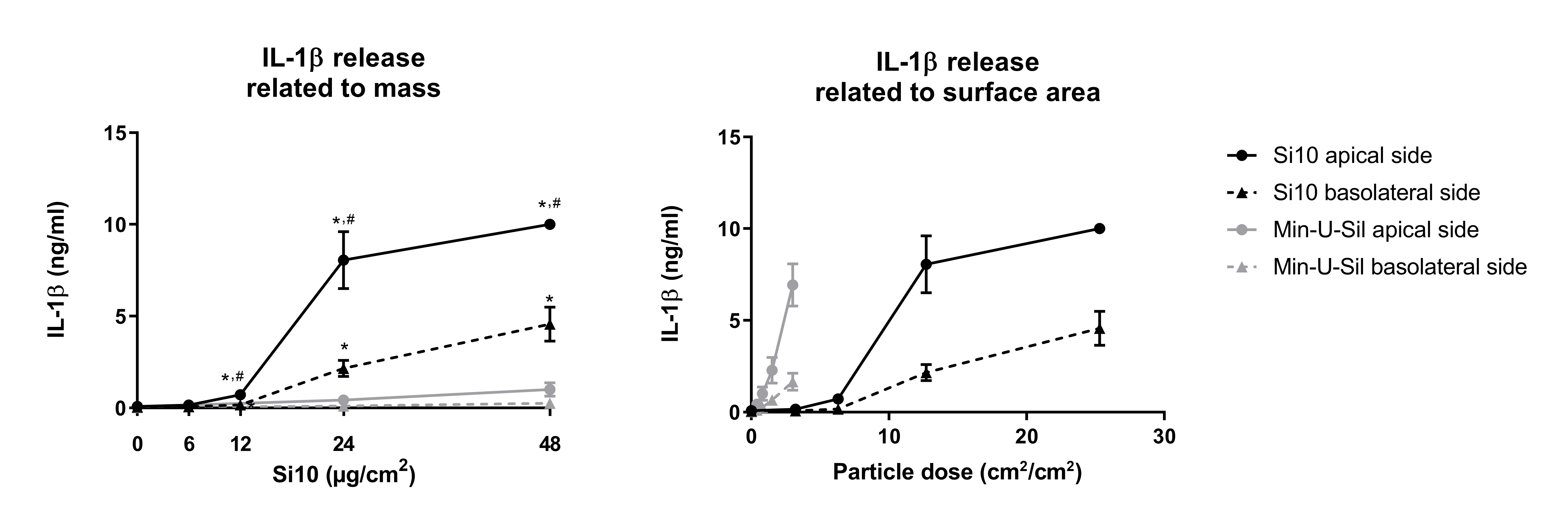

Supplement: Supplementary file 3 — Additional file 3: Figure S3. Concentration-dependent release of IL-1β after Si10 and Min-U-Sil exposure in 3D tri-culture related to particle mass and surface area. The figure shows cytokine levels in the apical and the basolateral compartment after 20 h exposure to 0–48 μg/cm2 Si10 and 0–192 μg/cm2 Min-U-Sil equivalent to 0–25 cm2/cm2 of Si10 and 0–3 cm2/cm2 of Min-U-Sil. Cytokine levels were determined by ELISA, and data represent the mean +/− SEM of 5 independent experiments. [file 12989_2020_345_MOESM3_ESM.tif]

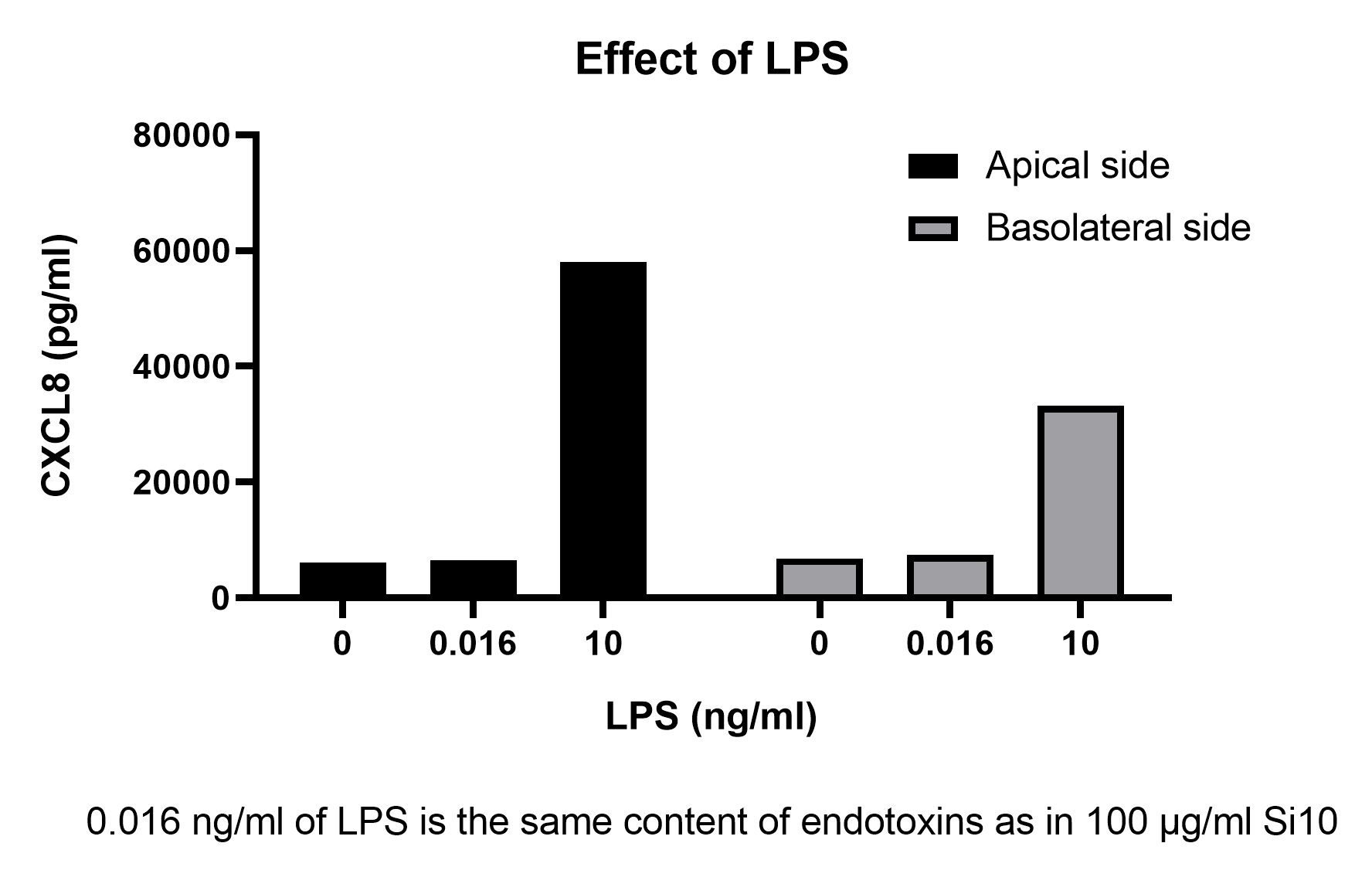

Supplement: Supplementary file 4 — Additional file 4: Figure S4. Effect of LPS on CXCL8 release in the 3D tri-culture. The 3D tri-culture consisted of THP-1 macrophages and A549 cells in the apical compartment and Ea.hy 926 endothelial cells in the basolateral compartment as described in Material and Methods. The figure shows the CXCL8 release in the apical and the basolateral compartment after 20 h exposure to LPS (0.016 and 10 ng/ml LPS) [file 12989_2020_345_MOESM4_ESM.tif]
